# Keystone-design perforator island flaps for the management of complicated epidermoid cysts on the back

**DOI:** 10.1038/s41598-019-51289-4

**Published:** 2019-10-11

**Authors:** Chi Sun Yoon, Hyo Bong Kim, Young Keun Kim, Hoon Kim, Kyu Nam Kim

**Affiliations:** 1 Department of Plastic and Reconstructive Surgery, Ulsan University Hospital, University of Ulsan College of Medicine, Ulsan, Korea; 2 Department of Plastic and Reconstructive Surgery, Konyang University Hospital, University of Konyang College of Medicine, Myunggok Medical Research Center, Daejeon, Korea; 3 Kim Young Keun’s Plastic and Aesthetic Surgery Clinics, Daejeon, Korea

**Keywords:** Cancer epidemiology, Cysts

## Abstract

Complicated epidermoid cysts (ECs) occur commonly on the back, but few reports have described their management. We present our experience in managing patients with ECs on the back using a keystone-design perforator island flap (KDPIF) reconstruction, thereby focusing on reduction and redistribution of wound tension. Altogether, 15 patients (average age, 48.067 ± 14.868 years) underwent KDPIF reconstructions after complete excision of complicated ECs on the back. We retrospectively reviewed the medical records and clinical photographs of all patients. Final scar appearance was evaluated using the Patient and Observer Scar Assessment Scale (POSAS). All patients had ruptured ECs, while 6 patients also had cellulitis of the surrounding tissues. All defects, after complete excision of ECs and debridement of surrounding unhealthy tissues, were successfully covered with KDPIF. The mean ‘tension-change’ at the defect and donor sites was −4.73 ± 0.21 N and −4.88 ± 0.25 N, respectively (p < 0.001). The mean ‘rate of tension-change’ at the defect and donor sites was −69.48 ± 1.7% and −71.16 ± 1.33%, respectively (p < 0.001). All flaps survived with no postoperative complications. The mean observer scar assessment scale (OSAS) summary score and patient scar assessment scale (PSAS) total score were 14.467 ± 5.069 and 15.6 ± 6.512, respectively. Overall, we suggest that KDPIF reconstruction is a good surgical modality for the management of complicated ECs on the back.

## Introduction

Epidermoid cysts (ECs) are common benign masses of the skin, and their excision is one of the most routinely performed surgical procedures^1^. Surprisingly, a detailed method for excision of these cysts has not been described in standard surgical textbooks, though several techniques have been enumerated and explained in previously published papers^1^. In general, the surgical alternatives for EC excision include puncture with aspiration, minimal excision surgery, and total excision surgery^2^. The former 2 techniques are suitable for small and uncomplicated ECs^2–5^. They have the advantage of minimal scar formation but carry the risk of incomplete removal and recurrence^1–5^. In case of large-sized and complicated ECs, including those that are ruptured or infected, a total excision procedure including complete removal of the cystic components and debridement of the surrounding abnormal tissues should be performed to prevent recurrence. In such cases, which require clearance of a significant volume of adjacent tissue, it is difficult to achieve primary closure of the resultant surgical wounds^6^. A basic principle of reconstructive surgery is to achieve wound closure with minimal tension along with obliteration of dead space. However, primary closure performed with a considerable amount of tension can result in persistent dead space formation owing to ineffective wound closure, which may lead to hematoma and seroma formation, wound dehiscence, delayed healing, and localised infection^6^. Several techniques, including undermining, imbrication, and creating various types of local flaps are utilised to achieve proper closure as per this principle^7–9^. While undermining or imbrication can achieve mild to moderate decrease in tension during wound closure along with minimal filling of dead space^9^, conventional local flaps, such as advancement-, rotation-, and transposition flaps not only allow for coverage of skin and soft tissue defects without tension, but also pack the dead space completely^6,9^. Recently, the keystone-design perforator island flap (KDPIF), a type of localised flap, has gained preference for reconstruction of various sites^10^. We therefore applied the technique of KDPIF reconstruction to the wounds following complete excision of complicated ECs, to prevent postoperative complications and recurrence. In this study, we present a retrospective review of our experience with using the KDPIF for reconstruction following management of complicated ECs occurring on the back. Our study especially focuses on evaluating the wound tension-reducing and -redistributing effects of the KDPIF, for which we compared ‘pre-flap wound tension’ with ‘post-flap wound tension’ using an intraoperative tensiometer.

## Results

**Figure 1:**
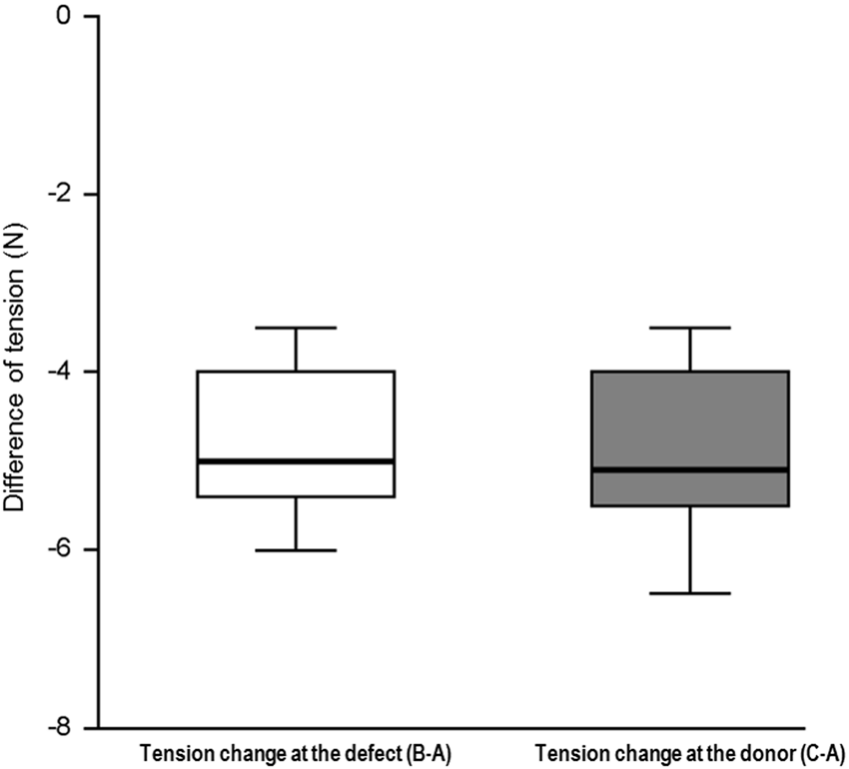
Comparison of mean differences between paired variables of the tensiometer data expressed as ¼ Q (first quartile), Median, ¾ Q (third quartile). A, pre-flap tension at the defect; B, post-flap tension at the defect; C, post-flap tension at the donor.

Table 1 summarises the patients’ characteristics and their clinical data. Tables 2, 3 show the data distribution of the recorded continuous and categorical variables, respectively. All patients included in the study had ruptured ECs with 6 patients suffering from an associated cellulitis of the surrounding tissues. Total 3 patients had undergone a previous operation including excision of the EC followed by primary closure but had subsequently developed postoperative wound dehiscence and delayed healing, thus requiring a secondary reconstruction procedure. Therefore, we performed a KDPIF reconstruction procedure after thorough debridement of the surrounding unhealthy tissues, for wound revision in all patients. The lesions were present on the upper, middle, and lower back in 10, 4, and 1 patient, respectively. The size of the defect varied from 1.5 × 2 cm to 3 × 8 cm (mean: 9.76 ± 6.189 cm). We constructed a Type IIA KDPIF in all patients with the flap sizes varying from 2 × 5 cm to 5 × 15 cm (mean: 21.9 ± 16.958 cm). Table 4 presents the tensiometer data and Table 5 shows the summary of variables of tension measurements. The mean values of A, B, and C were 6.91 ± 0.4 N, 2.18 ± 0.22 N, and 2.03 ± 0.18 N, respectively. The mean ‘tension-change at the defect’ (B−A) and ‘tension-change at the donor’ (C−A) sites were −4.73 ± 0.21 N and −4.88 ± 0.25 N, respectively (*p* < 0.001). The mean ‘rate of tension-change at the defect-’ $(\frac{{\rm{B}}-{\rm{A}}}{{\rm{A}}} \% )$ and ‘rate of tension-change at the donor-’ $(\frac{{\rm{C}}-{\rm{A}}}{{\rm{A}}} \% )$ sites were −69.48 ± 1.7% and −71.16 ± 1.33%, respectively (*p* < 0.001). Thus, the post-flap tension levels at both the defect- and donor sites were found to have significantly decreased as compared to the level of pre-flap tension at the site of the defect. Figure 1 shows the comparison of the mean differences between the paired variables of the tensiometer data. All the reconstructed flaps survived fully, and no postoperative complications, including wound dehiscence, hematoma, or seroma formation or recurrence of the EC occurred in any of the patients. Tables 6, 7 show the results of observer scar assessment scale (OSAS) and patient scar assessment scale (PSAS) assessments, respectively. The mean OSAS final score was 14.467 ± 5.069, and the mean objective scar rating was 3.467 ± 1.598. The mean PSAS total score was 15.6 ± 6.512, and the mean overall patient satisfaction rating was 3.8 ± 1.32.

**Table 1:** Patient characteristics and perioperative data.

Case	Sex/Age (yrs)	Diagnosis	Location	*Previous operation	Defect size (cm)	Flap size (cm)	Flap survival	Complication	Follow-up periods (months)
1	M/31	Ruptured EC with cellulitis	Middle right back	N	3 × 5	3 × 9	Fully survived	N	14
2	M/51	Ruptured EC	Upper left back	N	2.5 × 3	2.5 × 6	Fully survived	N	11
3	M/55	Ruptured EC	Upper left back	N	2 × 2.5	2 × 6	Fully survived	N	12
4	M/52	Ruptured EC with cellulitis	Upper right back	N	3.5 × 5	3.5 × 10	Fully survived	N	9
5	F/49	Ruptured EC	Upper midline back	Y	2.5 × 4	2.5 × 7.5	Fully survived	N	14
6	M/28	Ruptured EC with cellulitis	Upper midline back	Y	3 × 8	5 × 15	Fully survived	N	16
7	F/56	Ruptured EC	Upper midline back	N	2 × 2.5	2 × 5	Fully survived	N	10
8	M/30	Ruptured EC with cellulitis	Middle midline back	Y	2.5 × 3	2.5 × 5.5	Fully survived	N	14
9	M/29	Ruptured EC	Middle left back	N	2.5 × 3.5	2.5 × 6	Fully survived	N	12
10	M/60	Ruptured EC with cellulitis	Upper midline back	N	3 × 6	3.5 × 10	Fully survived	N	12
11	F/36	Ruptured EC	Upper right back	N	2 × 2.5	2 × 5	Fully survived	N	10
12	M/44	Ruptured EC	Upper right back	N	1.7 × 2	2 × 5	Fully survived	N	9
13	M/62	Ruptured EC	Middle midline back	N	1.5 × 2	2.5 × 6	Fully survived	N	9
14	F/59	Ruptured EC with cellulitis	Lower left back	N	3 × 3.5	3.5 × 7	Fully survived	N	11
15	M/79	Ruptured EC	Upper right back	N	2.5 × 2.5	2.5 × 5	Fully survived	N	12

*Previous operation means previous surgical management (excision of EC and primary closure) for ECs before keystone perforator island flap reconstruction.

EC, epidermoid cyst.

**Table 2:** Data distribution for continuous variables in patient characteristics.

Variable	Total N	Mean ± SD	Median	Min ~ Max Value	Q1 ~ Q3	IQR ( = Q3 - Q1)
Age (yrs)	15	48.067 ± 14.868	51	28 ~ 79	31 ~ 59	28
Defect size (cm)	15	9.76 ± 6.189	7.5	3 ~ 24	5 ~ 15	10
Flap size (cm)	15	21.9 ± 16.958	15	10 ~ 75	12 ~ 27	15
Follow-up period (months)	15	11.667 ± 2.127	12	9 ~ 16	10 ~ 14	4

Total N, total sample number; SD, standard deviation; Q1 ~ Q3, first quartile ~ third quartile; IQR, inter quartile range.

**Table 3:** Data distribution for categorical variables in patient characteristics.

Variable	Subgroup	N	%
Sex	Total	15	100
	Female	4	26.7
	Male	11	73.3
Diagnosis	Total	15	100
	Ruptured EC	9	60
	Ruptured EC with cellulitis	6	40
Location	Total	15	100
	Lower left back	1	6.7
	Middle left back	1	6.7
	Middle midline back	2	13.3
	Middle right back	1	6.7
	Upper left back	2	13.3
	Upper midline back	4	26.7
	Upper right back	4	26.7
Previous operation	Total	15	100
	No	12	80
	Yes	3	20
Flap survival	Total (fully survived)	15	100
Complication	Total (none)	15	100

EC, epidermoid cyst; N, sample number; %, percentage.

**Table 4:** Tensiometer data.

Case	A. Pre-flap tension at the defect (N)	B. Post-flap tension at the defect (N)	C. Post-flap tension at the donor (N)	Tension-change at the defect (B-A, N)	Tension-change at the donor (C-A, N)	Rate of tension-change at the defect (B-A/A%)	Rate of tension-change at the donor (C-A/A%)
1	7.5	2.5	2.3	−5	−5.2	−66.67	−69.33
2	8	2.3	2.3	−5.7	−5.7	−71.25	−72.25
3	8.5	3	2.7	−5.5	−5.8	−64.71	−68.24
4	7.6	2.5	2.5	−5.1	−5.1	−67.11	−67.11
5	9.5	3.5	3	−6	−6.5	−63.16	−68.42
6	8	3	2.5	−5	−5.5	−62.5	−68.75
7	5	1	1.5	−4	−3.5	−80	−70
8	6.5	2	1.5	−4.5	−5	−69.23	−76.92
9	8.1	2.7	2.7	−5.4	−5.4	−66.67	−66.67
10	8.5	3.5	3	−5	−5.5	−58.82	−64.71
11	5.5	1.5	1.5	−4	−4	−72.73	−72.73
12	5	1.5	1.5	−3.5	−3.5	−70	−70
13	5	1.2	1	−3.8	−4	−76	−80
14	6	1	1	−5	−5	−83.33	−83.33
15	5	1.5	1.5	−3.5	−3.5	−70	−70

N, Newton.

**Table 5:** Summary of the tensiometer data.

Variables	Mean ± SE	*p* value
Mean value of Pre-flap tension at the defect (A, N)	6.91 ± 0.4	NA
Mean value of Post-flap tension at the defect (B, N)	2.18 ± 0.22	NA
Mean value of Post-flap tension at the donor (C, N)	2.03 ± 0.18	NA
Mean value of tension-change at the defect (B-A, N)	−4.73 ± 0.21	<0.001
Mean value of tension-change at the donor (C-A, N)	−4.88 ± 0.25	<0.001
Mean value of Rate of tension-change at the defect (B-A/A%)	−69.48 ± 1.7	<0.001
Mean value of Rate of tension-change at the donor (C-A/A%)	−71.16 ± 1.33	<0.001

N, Newton; All variables, difference of paired variables, and rate of change were expressed as Mean ± SE, p value computed using Wilcoxon signed-rank test.

**Table 6:** Mean values of the Observer Scar Assessment Scale (OSAS).

	Mean ± SD
Vascularity	2.867 ± 0.99
Pigmentation	2.867 ± 1.125
Thickness	3.133 ± 1.246
Relief	2.733 ± 0.961
Pliability	2.867 ± 1.06
OSAS summary score	14.467 ± 5.069
Objective scar rating	3.467 ± 1.598

SD, standard deviation.

**Table 7:** Mean values of the Patient Scar Assessment Scale (PSAS).

	Mean ± SD
Is the scar painful?	1.467 ± 0.915
Is the scar itching?	2.333 ± 1.543
Is the colour of the scar different?	3.20 ± 1.32
Is the scar more stiff?	2.8 ± 1.082
Is the thickness of the scar different?	3 ± 1.309
Is the scar irregular?	2.8 ± 0.862
PSAS total score	15.6 ± 6.512
Overall patient satisfaction	3.8 ± 1.32

SD, standard deviation.

### Case presentations

#### Case 10

**Figure 2:**
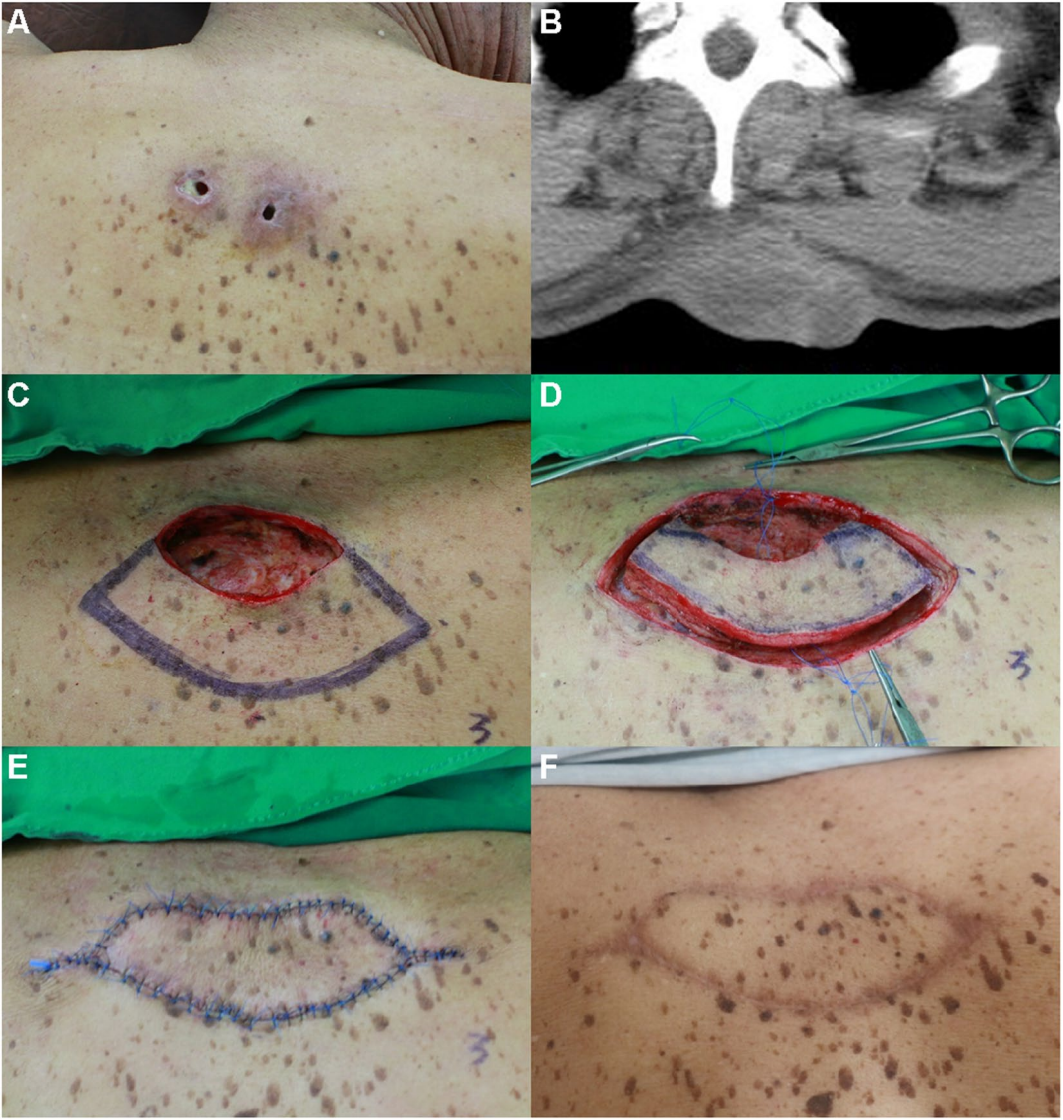
Keystone-design perforator island flap (KDPIF) reconstruction after complete excision of the ruptured epidermoid cyst (EC) with cellulitis in a 60-year-old man. (**A**) A 5-cm solid lesion having an indefinite border was palpated with two skin openings observed on the upper midline of the back. (**B**) Computed tomography scan revealed a ~7.1 × 3.4 × 3.3 cm multi-loculated cystic mass with wall enhancement, involving the skin and whole subcutaneous layer of the upper back. (**C**) The design of the flap. The final defect size was 3 × 6 cm and a 3.5 × 10 cm-sized KDPIF was designed in the lower side of the defect. (**D**) The island-form of the flap was made. (**E**) Postoperative clinical photograph immediately after the flap inset. (**F**) Postoperative clinical photograph of the flap after 12 months of follow-up.

A 60-year-old man visited our department with a painful mass associated with pus discharge located on his upper back along the midline (Fig. 2). The lesion had first presented ~1 year back and had gradually enlarged in size. The patient provided a history of squeezing the lesion several times to evacuate its contents, but it continued to enlarge. There was no previous history of surgery for the lesion. On palpation during physical examination, we found a solid lesion ~5-cm in size with an indeterminate border and two openings on its surface associated with pus-like discharge. Empirical antibiotic treatment (Flomoxef 1 g intravenous injection q12h) was administered for 1 week to relieve inflammation of the tissues surrounding the infected EC. A computed tomography (CT) scan of the lesion revealed a 7.1 × 3.4 × 3.3 cm-sized, multi-loculated cystic mass with wall enhancement, involving the skin and the entire subcutaneous layer of the upper back. The cystic mass was completely excised, and debridement of the surrounding unhealthy and adhesive tissues was carried out. The dimensions of the final defect were 3 × 6 cm. The ‘pre-flap tension at the defect’ was 8.5 N. A KDPIF of size 3.5 × 10 cm was designed from the lower side of the defect. The ‘post-flap tension at the defect-’ and ‘post-flap tension at the donor-’ sites were 3.5 N and 3.0 N, respectively. We achieved tension-free in-setting of the flap and primary closure of the donor site. The ‘tension-change at the defect-’ and ‘tension-change at the donor-’ sites were −5 N and −5.5 N, respectively. The ‘rate of change in tension at the defect-’ and ‘rate of change in tension at the donor-’ sites were calculated as −58.82% and −64.71%, respectively. The complete flap survived without any occurrence of postoperative wound complications. No recurrence was observed during the 12-month follow-up period. The OSAS summary score was 10, and the objective scar rating was 2. The PSAS total score was 15, and the overall patient satisfaction rating was 4.

#### Case 11

**Figure 3:**
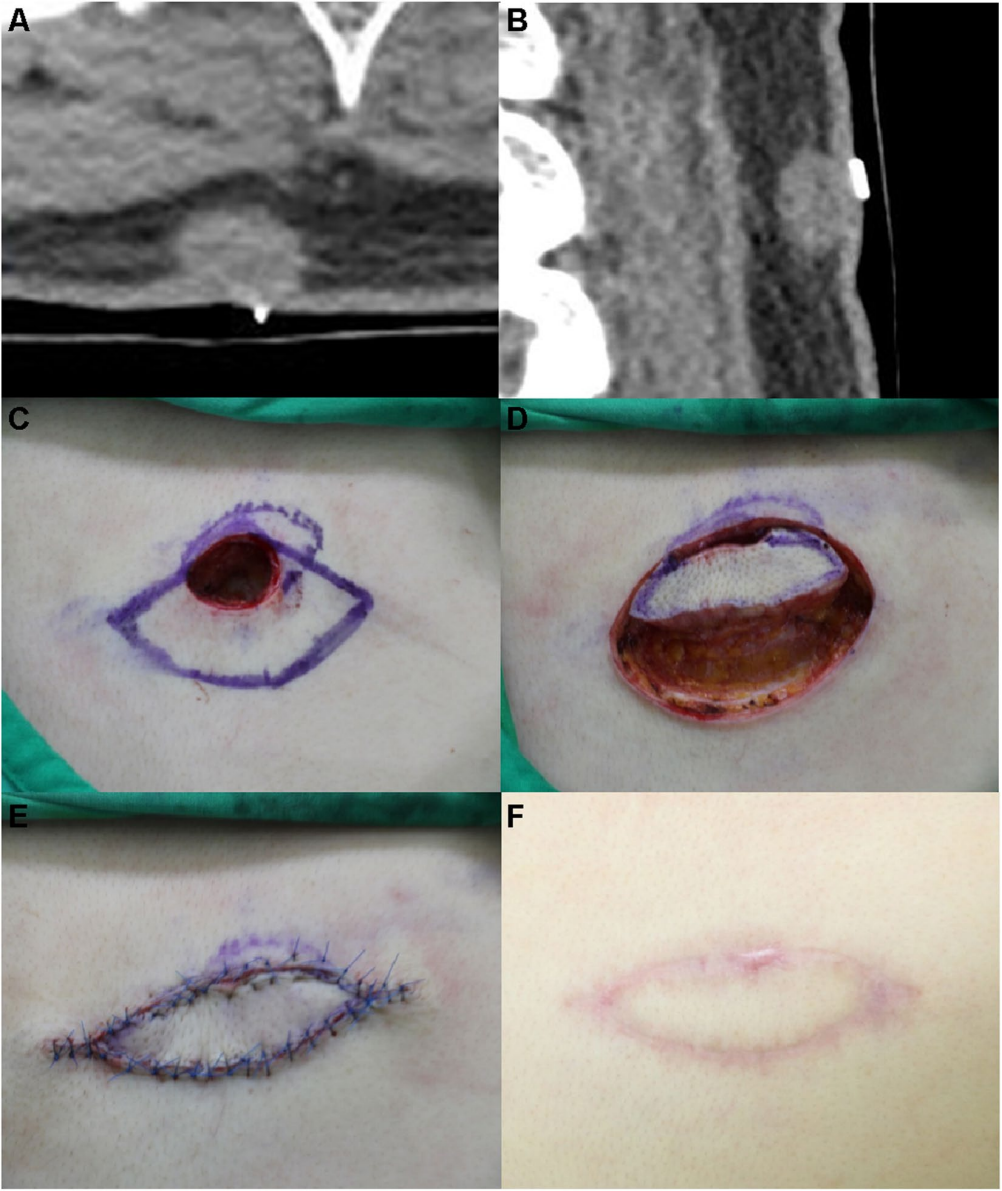
Keystone-design perforator island flap (KDPIF) reconstruction after complete excision of the ruptured epidermoid cyst (EC) in a 36-year-old woman. (**A**,**B**) Computed tomography scan revealed a 1.5 × 2 cm elliptical cystic lesion extending from the skin to the deep fat layer at the level of thoracic inlet. (**C**) The design of the flap. The final defect size was 2 × 2.5 cm and a 2 × 5 cm-sized KDPIF was designed in the lower side of the defect. (**D**) The island-form of the flap was designed and the flap was moved to the defect with minimal tension. (**E**) Postoperative clinical photograph immediately after the flap inset. (**F**) Postoperative clinical photograph of the flap after 10 months of follow-up.

A 36-year-old woman visited our department with a solid lesion on her right upper back, which had first presented about 1.5 years ago and had gradually enlarged in size (Fig. 3). She had squeezed the lesion several times to evacuate it, but the lesion continued to enlarge. There was no previous history of operative management of the lesion. On physical examination, we could palpate a ~2-cm mass having an indeterminate border with a single pinpoint skin opening. There were no signs of inflammation of the surrounding tissues. The CT scan revealed a 1.5 × 2 cm elliptical cystic lesion extending from the skin to the deep subcutaneous fat layer at the level of thoracic inlet. We excised the cystic mass entirely and performed debridement of the surrounding tissue adhesions. The size of the final defect was 2 × 2.5 cm, and the ‘pre-flap tension at the defect site’ was 5.5 N. A KDPIF of size 2 × 5 cm was designed from the lower side of the defect. The ‘post-flap tension at the defect-’ and ‘post-flap tension at the donor-’ sites was 1.5 N each, respectively. A tension-free in-setting of the flap and primary closure of the donor site were performed. The value of ‘tension-change at the defect-’ and ‘tension-change at the donor-’ sites was −4 N each. The ‘rate of tension-change at the defect-’ and ‘rate of tension-change at the donor-’ site were −72.73% and −72.73%, respectively. The entire flap survived with no postoperative wound complications. No recurrence was observed during the 10-month follow-up period. The OSAS final score was 12, and the objective scar rating was 3. The PSAS total score was 13, and the overall patient satisfaction rating was 3.

#### Case 14

**Figure 4:**
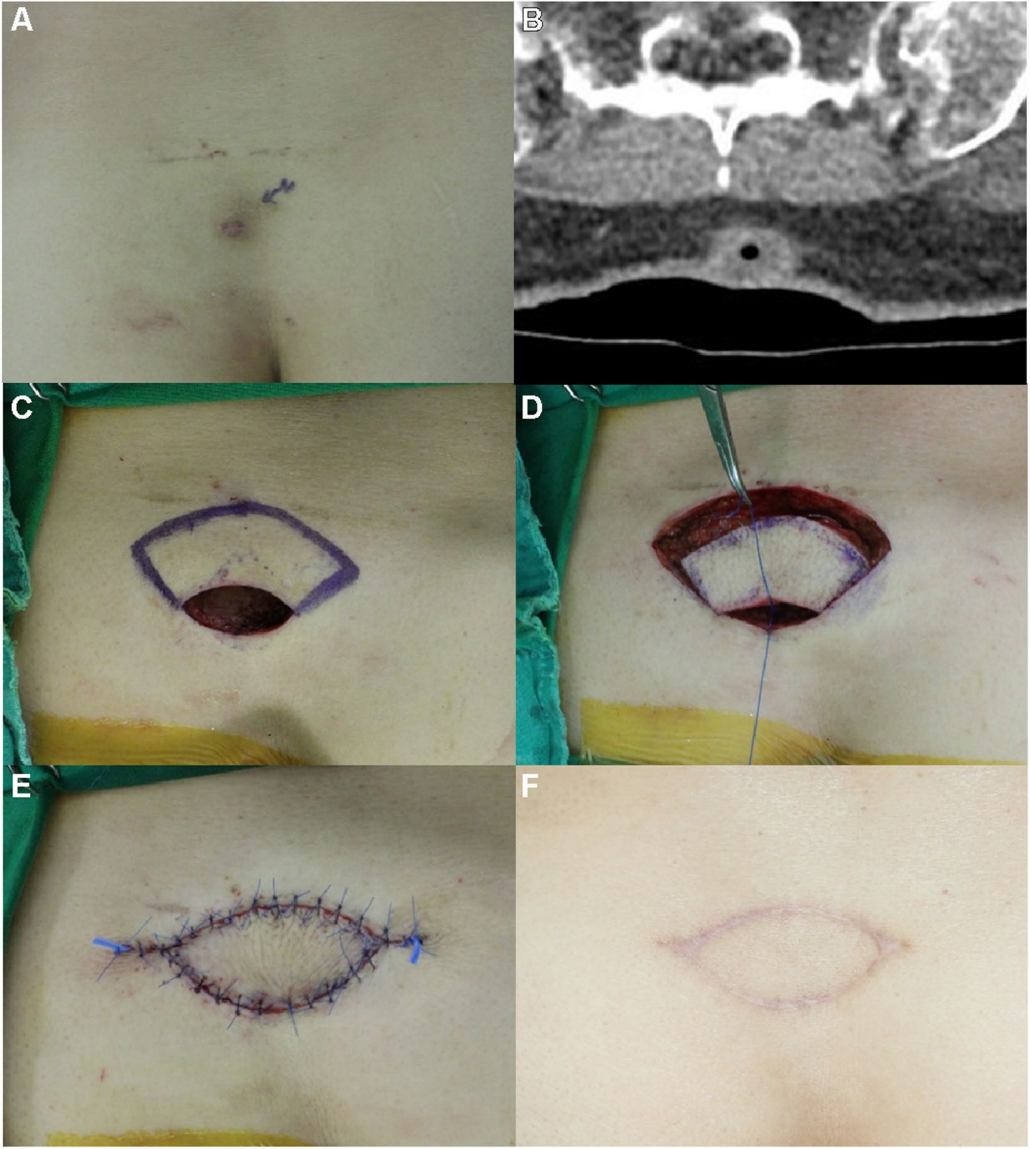
Keystone-design perforator island flap (KDPIF) reconstruction after complete excision of the ruptured epidermoid cyst (EC) with cellulitis in a 59-year-old woman. (**A**) A 3-cm-sized mass-like lesion having unclear border was palpated on her lower left back after administering antibiotic treatment for 1 week. (**B**) Computed tomography scan revealed a 1.5 × 1.8 × 2.5 cm-sized enhancing cystic lesion with inner air from skin to deep fat layer at the level of left upper sacral border. (**C**) The design of the flap. The final defect size was 3 × 3.5 cm, and a 3.7 × 7 cm KDPIF was designed on the upper side of the defect. (**D**) The island-form of the flap was created, and the flap was moved to the defect with minimal tension. (**E**) Postoperative clinical photograph immediately after the flap inset. (**F**) Postoperative clinical photograph of the flap after 11 months of follow-up.

A 59-year-old woman visited our department with a painful, solid EC with associated discharge on her left lower back (Fig. 4). The lesion had first developed ~3 years ago and had gradually enlarged. She had squeezed the lesion many times, but the lesion continued to grow. She had visited a local clinic and undergone incision and drainage of the EC several times. However, the lesion had persisted and increased in size. On palpation during physical examination, we found a 3-cm mass with an undefined border and pinpoint skin opening associated with the discharge. As the surrounding tissues were inflamed, empirical antibiotic treatment was administered for 1 week. A subsequent CT scan revealed an enhancing cystic lesion of size 1.5 × 1.8 × 2.5 cm containing inner air pockets and extending from the skin surface to the deep fat layer at the level of left upper sacral border. We excised the entire cystic mass and performed debridement of the surrounding unhealthy tissue and adhesions. The final defect was found to be 3 × 3.5 cm in size, and the ‘pre-flap tension at the defect site’ was 6.0 N. We designed a KDPIF of size 3.5 × 7 cm from the upper side of the defect. The ‘post-flap tension at the defect-’ and ‘post-flap tension at the donor-’ sites was 1.0 N each, respectively. We achieved tension-free in-setting of the flap and primary closure of the donor site. The ‘tension-change at the defect-’ and ‘tension-change at the donor-’ site was −5 N and −5 N, respectively. The rate of change in tension at the defect- and the donor site was 83.33% each. The flap survived completely with no postoperative wound complications. No recurrence was observed during the 11-month follow-up period. The OSAS summary score was 10, and the objective scar rating was 2. The PSAS total score was 12, and the overall patient satisfaction score was 3.

## Discussion

We retrospectively reviewed the medical records and clinical photographs of all patients who underwent KDPIF reconstruction of defect following excision of EC and evaluated the final scar appearance using the POSAS. All flaps survived with no postoperative complications. OSAS summary score and PSAS total score were 14.467 ± 5.069 and 15.6 ± 6.512, respectively.

The surgical treatment of uncomplicated ECs is simpler than that of the complicated cysts. The cystic wall in the former is well maintained and less adherent to surrounding tissues than that in complicated ECs, making dissection and removal of the uncomplicated cysts from surrounding tissues easier. However, in complicated ECs, including those that are ruptured, infected, or recurrent, most of the cystic wall adheres to surrounding tissues, which makes its complete separation from the tissue bed difficult^6^. Furthermore, the surrounding tissues are frequently unhealthy or devitalised and may need additional debridement^6^. Therefore, complete excision of complicated ECs should include removal of the entire cystic contents along with debridement of the infected and adhesive tissues surrounding the cyst to prevent infection and recurrence and to promote wound healing^6^. Consequently, the complete excision of complicated ECs can result in larger tissue defects than those occurring after excision of uncomplicated cysts, which precludes achieving primary closure with minimal wound tension of the former defect^6^. Moreover, immoderate primary wound closure can cause wound dehiscence and delay healing^6^.

While complicated ECs commonly occur on the back, there have been few published reports on the topic, and their management has not been described in the existing literature. The back is covered with thicker and stiffer skin^14^, which can sustain greater tension during body movements than the thinner integument covering other regions of body, such as the face and neck^14,15^. Therefore, it is very important to repair wounds on the back with minimal tension to achieve successful healing. Operative management of complicated ECs not only involves removal of cysts, but also includes debridement of surrounding tissues along with co-excision of the elliptical skin covering the cyst, which results in significant soft tissue defects. Considering this and the inherent characteristics of skin over the back, primary wound closure should be performed carefully, especially after complete excision of complicated ECs occurring in this region. In our study, 3 patients underwent EC excision with primary closure but experienced recurrent postoperative wound complications, and therefore, needed a secondary reconstructive procedure. In these cases, primary closure at the first operation might have been ineffective for minimising wound tension against the skin stiffness of the back and might not have withstood the tension during back movement. Consequently, it resulted in the formation of persistent dead space, delayed wound healing, and wound dehiscence. KDPIF reconstruction following debridement of the surrounding unhealthy tissues in all 3 patients achieved complete flap survival and wound healing with no complications. Consequently, our experience with these cases suggests that the primary closure after removal of the complicated ECs occurring on the back, should be performed cautiously, and local flap coverage should be seriously considered as a part of the operative management of these cysts. Meanwhile, there was no cut-off for the size of the cyst for to undergo keystone flap reconstruction in the present study. The smallest defect among our cases was 1.5 × 2 cm, which could be considered adequate to achieve primary closure with undermining or imbrication. However, properties of the skin may differ from case to case; therefore, some small defects might have achieved primary closure with some wound tension. We considered that individual skin properties of the patient and characteristics of surrounding tissues may be crucial factors in determining the efficacy of flap reconstruction for defect coverage. In the present study, we performed keystone flap reconstruction in all cases because we believed that it would be effective in minimising wound tension in the back and withstanding tension during movements of the back, which would facilitate complete wound healing without complications.

The KDPIF devised by Behan in 2003, a multi-perforator-based fasciocutaneous island flap with two conjoined V-Y flaps^16^, is now commonly utilized for covering cutaneous defects occurring in various regions owing to its simple, defect-adaptive design, easy reproducibility, safety, and short operative time^10,17,18^. It can be used for covering not only small to moderate defects, but also for larger defects, for which free flap coverage may be required^19,20^. Despite its versatility and effectiveness, some authors, based on their experiments with ‘fresh-frozen’ cadavers, have contended that the keystone flap closure has no clear biomechanical rationale for reduction of wound tension and have questioned the ability of expansion of the flap skin-paddle^21,22^. However, our study showed that the levels of post-flap tension at both the defect and donor sites were significantly decreased compared with the level of pre-flap tension at the site of the defect, which indicated that the keystone flap had certainly reduced wound tension. The measurements and outcomes of our study were obtained from real patients (human bodies) unlike those derived using fresh-frozen cadavers, as in the previous study^21,22^. Furthermore, the operating surgeon can easily appreciate the release of tension surrounding the flap during stepwise dissection of the skin and fascia while constructing the island flap. Therefore, the flap can be advanced to the defect with minimal tension. Shayan *et al*. emphasised the principle of recruitment of laxity in KDPIF reconstruction, based on which a soft tissue defect in an area without surrounding tissue laxity is exchanged for a secondary defect in an adjacent area that does have sufficient laxity to enable primary closure^23^. They described how the V-Y advancement flaps at either end of the KDPIF facilitate this recruitment of laxity and the skin tension is redistributed perpendicular to the line of advancement, i.e., into the direction of maximal wound tension^23^. Based on this principle and the results of our study, we consider that the KDPIF reconstruction of the skin covering the back is effective in reducing and redistributing wound tension. Furthermore, KDPIF reconstruction after excision of the complicated ECs on the back may facilitate wound healing by reducing wound tension (redistribution of tissue laxity), filling up of the persistent dead space (volumetric effect), and improving flap vascularity (can be correlated with an observation of flap hyperaemia or hyper-perfusion also described as the ‘red-dot’ sign)^23^.

In summation, the goal of surgery for complicated ECs is postoperative wound healing without complications and recurrence of the cyst, which can be achieved by complete removal of the cyst, debridement of the surrounding unhealthy tissue, and a tension-free wound closure. We performed CT or ultrasonography (US) scans, which are covered by our national medical insurance system, for all patients in this study to evaluate the dimensions of the ECs and the condition of the surrounding tissues. Therefore, the extent of excision of the lesions was determined based on the findings to achieve complete excision of the EC. Thereafter, we covered the resultant defect on the back with a KDPIF, which distributes the tension evenly around the whole wound, thereby decreasing the chances of wound complications, thus promoting healing.

We did not use a hand-held ultrasound Doppler device to detect the presence of perforators in all our cases. We considered it unnecessary to locate the perforators in KDPIF reconstruction performed on the back because the integument covering this region has plenty of perforators and vascular connections from the segmental posterior intercostal, subcostal, and lumbar arteries^24^. In addition, the technique of minimal undermining of the flap in KDPIF construction guarantees stable flap perfusion and vascularity^10,25^. This, we believe, is the reason for survival of the reconstructed flaps in all patients in our study without a prior confirmation of the presence of flap-perforators using ultrasound.

Although we successfully managed complicated ECs using KDPIF reconstruction, our study has several limitations. Firstly, using this method, an extension of the operative scar is inevitable as compared to the linear scar occurring after primary closure with undermining or imbrication, followed by complete wound healing and no recurrence. Secondly, the skin among Asians with its unique characteristics of thicker dermis, more sebaceous glands, and increased melanin production demonstrates a tendency toward hyperpigmentation and scar formation after skin injury, which results in prolonged erythema during scar maturation and hypertrophic scar formation^26–28^. Although we recommended the use of a self-adherent soft silicone sheeting for 5 months to all patients in this study, development of hypertrophic scars was observed in 4 patients, affecting the mean OSAS score and mean objective scar rating, which were 14.467 ± 5.069 and 3.467 ± 1.598, respectively. Therefore, a thorough explanation of the possibility of scarring should be given to each patient preoperatively to impress the necessity for prolonged postoperative scar management. Finally, our study is a retrospective, non-randomised study with no comparison group and includes a relatively small number of cases. Further, our cases were not homogenous and included those that underwent primary and secondary reconstruction. Therefore, occurrence of selection bias and the presence of confounding factors was unavoidable and may have affected the outcome. Future large-scale prospective studies that include homogenous cases with group comparisons for different types of procedures, including undermining, imbrication, and other local flap techniques are warranted to validate our findings.

## Methods

This study was approved by the institutional ethics review board of the Konyang University Hospital (approval number: KUH 2019-02-014) and was conducted according to the ethical guidelines of the 1975 Declaration of Helsinki. All patients in this study provided written informed consent.

Between May 2016 and August 2018, 15 patients (11 men, 4 women), with an average age of 48.07 years (range: 29–79 years) underwent KDPIF reconstruction after complete excision of complicated EC presenting on the back. We retrospectively reviewed data including the locations of the ECs, whether previously operated, the size of the defects, flap sizes used, ‘pre-flap-’ and ‘post-flap tension’ levels at the defect, ‘post-flap tension’ at the donor site, flap survival, complications, and follow-up periods for each patient. The POSAS, which is a reliable and feasible tool for linear scar evaluation^11–13^, was used for assessing the final scar appearance in this study. A single observer (corresponding author, Kyu Nam Kim) estimated the OSAS score, which includes 5 items graded on a 10-point scale with 1 indicating normal skin and 10 indicating the worst scarring imaginable. The summary result score varied from 5 for normal skin to 50 for the worst possible scar. At the final follow-up, all patients self-assessed their scars using the PSAS score, which includes 6 items graded on a 10-point scale. The final score varies from 6 for normal skin to 60 for the worst imaginable scar. After scoring the items, both the observer and patient rated the overall scar appearance as ‘objective scar rating’ and ‘overall patient satisfaction’, respectively, based on a visual analogue scale corresponding to a 10-point scale (excellent to poor),^13^.

### Preoperative managements

In case of an EC associated with inflammation of surrounding tissues (cellulitis), empirical antibiotic treatment was administered until the infection subsided. All patients underwent preoperative imaging with CT or US to delineate the size and depth of the EC, degree of adhesion to surrounding tissues, and condition of the surrounding tissue and to rule out other pathologies. We planned the extent of excision of the lesion in each patient based on the CT scan or US findings.

### Surgical techniques

**Figure 5:**
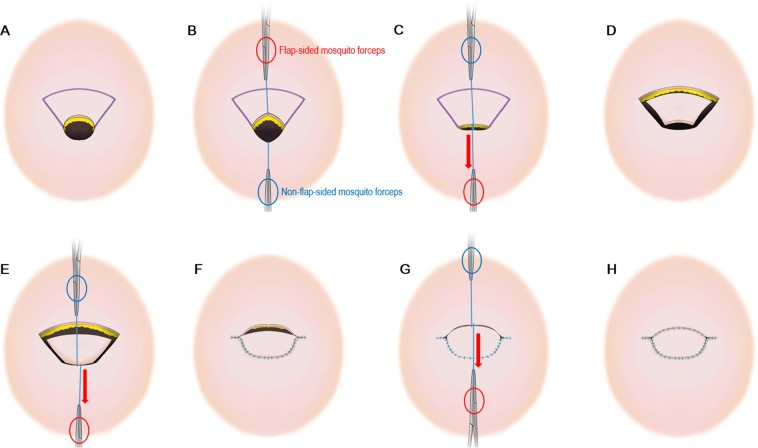
Schematic diagram of the keystone-design perforator island flap (KDPIF) and tension measurement procedures performed during excision of the epidermoid cyst on the back. Red-coloured oval represents the flap-sided mosquito forceps, and blue-coloured oval represents the non-flap-sided mosquito forceps. (**A**) Design of the KDPIF after complete excision of the complicated epidermoid cyst (EC). (**B**, **C**) Measurement of the ‘pre-flap tension at the defect’. (**D**) Complete elevation of the KDPIF, which is fully released and detached from the surrounding tissues. (**E**) Measurement of the ‘post-flap tension at the defect site’. (**F**) Defect-side flap closure achieved. (**G**) Measurement of the ‘post-flap tension at the donor site’. (**F**) Donor-side flap closure achieved.

Figure 5 presents a stepwise schematic diagram of the KDPIF creation and of the procedure for measuring tension. The operations were performed under local anaesthesia with the patients placed either in the prone or lateral decubitus position. We first excised the complicated EC completely, including the cystic mass components, followed by debridement of unhealthy surrounding tissues and adhesions. After complete excision, we recorded the size of the final defect, and the KDPIF was designed at either the upper or lower side of the defect based on tissue laxity. The long axis of the flap was created transversely, parallel to the relaxed skin tension lines (RSTLs) of the back, and the width of the flap was designed to be equal to or slightly larger than the width of the defect. Then, the tension across the most concave point of the wound border was measured with a tensiometer (Analog Force Gauge, Wenzhou Tripod Instrument Manufacturing Co., Ltd, Zhejiang, China). A 3-0 (Ethilone) suture was passed through both wound edges at the most concave points (i.e., across the widest portions of the defect) and a mosquito forceps was attached to each suture. The non-flap-sided forceps was held in place without pulling because it was only reference point for the direction of the flap-sided forceps. The flap-sided forceps was pulled apart gently until the wound edges nearly approximated with each other. Thereafter, the tensiometer was connected to a ring of the flap-sided mosquito forceps to record wound tension. Three consecutive measurements were performed, and the average value was used to indicate the ‘pre-flap tension at the defect’ (Fig. 5C).

**Figure 6:**
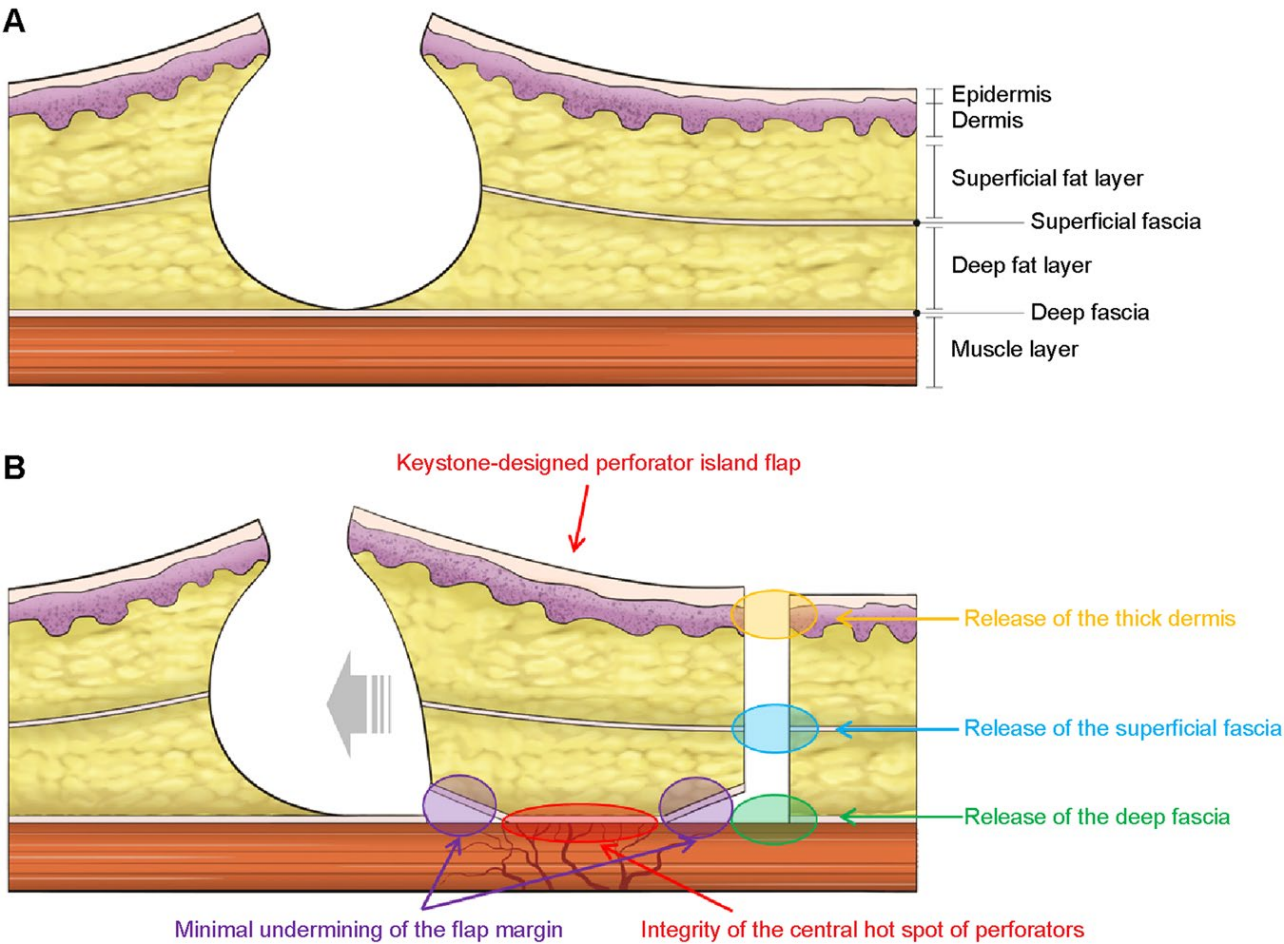
Schematic diagram of the keystone-design perforator island flap (KDPIF) movement. (**A**) Cross-sectional diagram after complete excision of the complicated epidermoid cyst (EC). (**B**) The KDPIF movement on the back is provoked by three main factors. The first factor (orange-coloured oval) is the release of the thick dermis of back; the second (blue-coloured oval) is the release of the superficial fascia; and the last (green-coloured oval) is the release of the deep fascia. Then, minimal flap undermining of the flap margin (purple-coloured circle) is performed to preserve the integrity of the ‘central hot spot’ of perforators (red-coloured oval).

**Figure 7:**
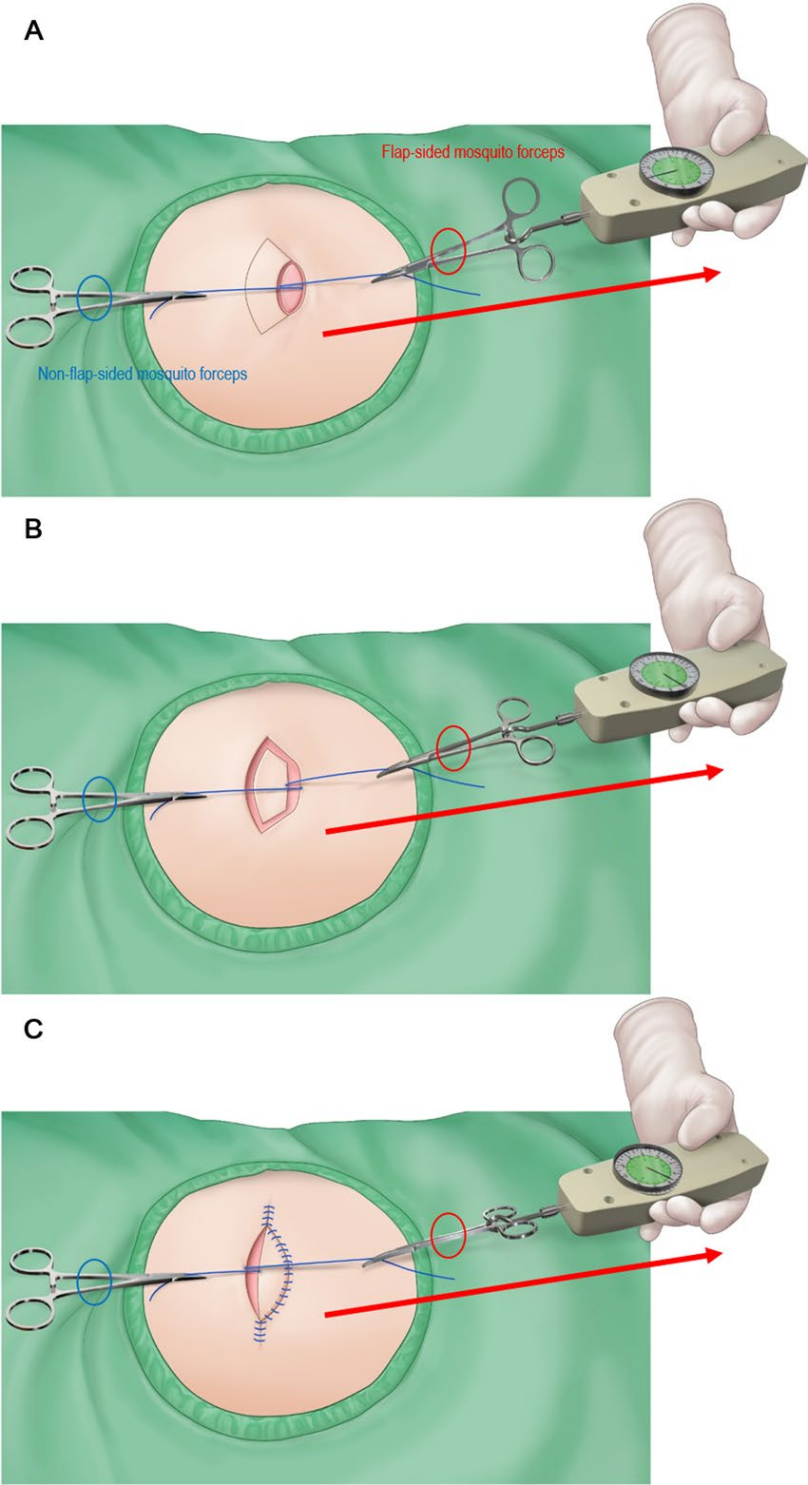
Schematic illustration of the procedure for intraoperative tensiometer measurement. A 3-0 (Ethilone) suture is passed through both wound edges at the most concave point (i.e., across the widest part of the defect), and a mosquito forceps is attached to each suture. The non-flap-sided forceps is kept in place without pulling because it is the only reference point for the direction of the flap-sided forceps. The flap-sided forceps is pulled apart gently until the wound edges are nearly approximated with each other. Then, the tensiometer is connected to a ring on the flap-sided mosquito forceps to record wound tension. Three consecutive measurements are performed, and the average value is used. Tensiometer is sterilised for intraoperative use. The red-coloured oval represents the flap-sided mosquito forceps, and the blue-coloured oval represents the non-flap-sided mosquito forceps. (**A**) Measurement of the ‘pre-flap tension at the defect’. (**B**) Measurement of the ‘post-flap tension at the defect’. (**C**) Measurement of the ‘post-flap tension at the donor’.

The skin incision was made along the flap, and we dissected the tissue from the subcutaneous layer to the deep thoracolumbar fascia. During dissection, the operating surgeon observed a gradual slack in the tension surrounding the flap, possibly owing to a sequential release of the thick dermis of back, followed by that of the superficial and deep fascia (Fig. 6). After creating the island-shaped flap structure, minimal undermining (up to 10 mm from the flap margin considering the flap movement and size) was performed to preserve the vascular integrity of the central hot spot (perforators). Then, the ‘post-flap tension at the defect’ site was measured with the tensiometer in the same manner as described above (Fig. 5E). The procedure of in-setting the flap was first performed at its central portion on the side of the defect, and then on both ends, which were aligned in a V-Y apposition. It is most important to suture the flap layer-by-layer on the defect-side during closure to avoid dead space formation. The ‘post-flap tension at the donor’ site was measured with the tensiometer (Fig. 5G) before closure of the flap on the donor-side. The donor site was then closed primarily and a mild compression dressing with foam material was applied. Figure 7 presents a schematic illustration of the intraoperative procedure for measurement of tension using the tensiometer.

### Postoperative managements

All patients were followed-up routinely in the outpatient clinic every 3~4 days for fresh dressing and wound assessment. The skin sutures were removed after 14 postoperative days, and steri-strips (3 M, Maplewood, MN) were applied for wound closure for 4 weeks in all patients to prevent wound dehiscence and scar widening. We also recommended that the patients use Mepiform (Mölnlycke Health Care, Oakville, Ontario, Canada), a self-adherent soft silicone sheeting designed for scar management, for further 5 months.

### Statistical analysis

#### Software and Basic Statistics

The R language version 3.3.3 (R Foundation for Statistical Computing, Vienna, Austria) software and the T&F program ver. 2.9 (YooJin BioSoft, Korea) were used for all statistical analyses. Continuous variables were expressed as mean ± SD (standard deviation), median, and IQR (inter quartile range). Categorical variables were expressed as sample number and percentage, N (%).

#### Comparison of mean difference of paired variables

The differences between paired variables such as the ‘pre-flap tension at the defect (A)’ and the ‘post-flap tension at the defect (B)’ or between the ‘pre-flap tension at the defect (A)’ and the ‘post-flap tension at the donor (C)’ were expressed as mean ± SE (standard error). The Wilcoxon signed-rank test was used to evaluate whether the mean difference between the paired variables was zero. The significance level was set at a *p* value of < 0.05.

## Conclusions

We successfully managed patients with complicated ECs on the back with excision and KDPIF reconstruction, without postoperative complications or recurrence. We also verified the tension-reducing effect of the KDPIF by comparing the pre-and post-reconstruction levels of wound tension. Therefore, we suggest that KDPIF reconstruction is a good surgical modality for the management of complicated ECs on the back. Based on our findings, the KDPIF reconstruction of complicated EC on the back can be summarised as follows: (1) Preoperative empirical antibiotic treatment should be administered in all patients having ECs with cellulitis; (2) Preoperative CT scan or US examination is recommended to plan the extent of excision and debridement; (3) Complete excision should include excision of all cystic components along with the debridement of surrounding unhealthy tissues and adhesions; (4) KDPIF can be designed at either the upper or lower side of the defect considering tissue laxity and should be constructed parallel to the RSTLs of the back to achieve a tension-free closure; and (5) Postoperative scar management should be recommended to patients for at least 5 months.

## Data Availability

All data generated or analysed during this study are included in this published article.
